# PREPARING TO MEET THE NEEDS OF OLDER ADULTS: WORKFORCE, TRAINING, AND EDUCATION ISSUES

**DOI:** 10.1093/geroni/igad104.1029

**Published:** 2023-12-21

**Authors:** Grace Caskie

## Abstract

Due to projected growth in the older adult population in the United States, those working in healthcare and education will encounter increasing numbers of older adults. This population shift presents challenges and opportunities for individuals working in these settings to be ready to meet the needs of older adults. In this symposium, we highlight research on several issues critical to preparing the workforce in healthcare and education for older adults’ specific needs. Poon’s paper addresses geriatric behavioral health workforce needs identified by a survey of licensed behavioral health providers. Several barriers to providing care to older adults are identified by these providers. Rodriguez et al. investigated the clinical practice experiences and challenges from ten years’ worth of alumni of the VA Geriatric Scholars program. This training program provides a model for improving clinical practice with older adults. Hollis-Sawyer examines older students’ first experiences taking a course in an online teaching setting and provides specific suggestions to maximize their learning environment. Patterson et al. present an extensive scoping review of end-of-life training for medical students that identified several themes that can direct efforts to improve end-of-life care for older adults. Finally, Caskie et al. address health biases and age biases in doctoral psychology trainees’ anticipated clinical work with depressed older adult clients of varying health statuses and provide guidance to psychology programs for improving training. This symposium aims to provide those working in healthcare and educational settings with ways to address workforce preparedness to meet the needs of older adults.

